# Ten-Month-Old Infants’ Reaching Choices for “more”: The Relationship between Inter-Stimulus Distance and Number

**DOI:** 10.3389/fpsyg.2013.00084

**Published:** 2013-03-07

**Authors:** Claudia Uller, Callum Urquhart, Jennifer Lewis, Monica Berntsen

**Affiliations:** ^1^School of Psychology, Criminology and Sociology, Kingston University, Kingston-upon-ThamesSurrey, UK; ^2^Faculty of Education, University of CambridgeCambridge, UK; ^3^Department of Psychology, University of EssexEssex, UK

**Keywords:** density assessment, number, infancy, representation

## Abstract

Animals and human infants discriminate numerosities in visual sets. Experiments on visual numerical judgments generally contrast sets in which number varies (e.g., the discrimination between 2 and 3). What is less investigated, however, is set density, or rather, the inter-stimulus distance between the entities being enumerated in a set. In this study, we investigated the role of set density in visual sets by 10-month-old infants. In Experiment 1, infants were offered a choice between two sets each containing four items of the exact same size varying in the distance in between the items (ratio 1:4). Infants selected the set in which the items are close together (higher density). Experiment 2 addressed the possibility that this choice was driven by a strategy to “select all in one go” by reducing the size and distance of items. Ten-month-olds selected the sets with higher density (less inter-stimulus distance) in both experiments. These results, although bearing replication because of their originality, seem consistent with principles in Optimal Foraging in animals. They provide evidence that a comparable rudimentary capacity for density assessment (of food items) exists in infants, and may work in concert with their numerical representations.

## Introduction

From neonates and pre-crawling babies, to toddlers and preschoolers, research indicates that numerical representations may be found early in development. While some researchers reach consensus that numerical understanding exists early in infancy, perhaps even innately (Uller et al., 1999; Feigenson et al., 2004; Cordes et al., 2007; Spelke and Kinzler, 2007; Cordes and Brannon, 2008; Uller, 2008), others prefer to claim that most numerical representations in children develop as a function of learning (e.g., Piaget, 1952). Another point of contention regards the nature of these abilities. Some argue that such representations may be conceptual (e.g., Carey, 2009), while others would prefer to link these abilities to perceptual cues (Clearfield and Mix, 2001; Cohen and Marks, 2002; Clearfield and Westfahl, 2006). Although several studies have used stimuli in a more “abstract” format to show that infant numerical abilities cannot be explained solely on the basis of a perceptual mechanism (auditory sets: Lipton and Spelke, 2003; events: Wynn, 1996; Wood and Spelke, 2005; cross-modal: Starkey et al., 1983; Feigenson et al., 2002; Kobayashi et al., 2004; Kobayashi et al., 2005; Uller, in preparation), researchers do not unanimously agree that these results may be indicative of a rudimentary form of numerical representations.

Another area of investigation to challenge the perceptual-only hypothesis comes from evidence that infants can approximate the number of items in large sets (e.g., visual objects: Xu and Spelke, 2000; Brannon, 2002; Brannon et al., 2004; puppet jumps: Wood and Spelke, 2005; auditory sets: Lipton and Spelke, 2003). For example, given a choice between two quantities, 8 versus 16, infants will discriminate between the two sets, either whether habituated to 8 or 16. These findings show that infants are not only able to represent small sets, but are also able to discriminate large sets, an ability which requires, at a very minimum, a representation of amount.

Control experiments contrasting number with variables such as cumulative surface area, perimeter contour, etc., also represent a challenge to perceptual explanations. Most of the number studies with infants control for various continuous variables, and the results suggest that the discriminations infants make are based on number (e.g., Xu and Spelke, 2000; Lipton and Spelke, 2003; Brannon et al., 2004; Xu and Arriaga, 2007; Cordes and Brannon, 2008). Cordes and Brannon (2008), for example, showed six-month-old infants conditions where cumulative surface area remained constant but number varied in a visual discrimination task. The babies detected the changes based on number rather than tracking total continuous extent of stimulus surface area.

While there is an emerging bulk of evidence for the number argument, contention with regards to variables which confound with number still persists. An aspect of visual sets controlled for is inter-stimulus distance, or set density. The adult visual perception literature on the relation between number and density seems to indicate that, in adults, estimated numerosity and density are negatively correlated (Krueger, 1972, 1984; Allik and Tuulmets, 1991, 1993; Durgin, 1995). For example, in the perception of dot patterns, dots are understood as less numerous when bunched together than when spread out (Krueger, 1972). Similarly, when judging relative numerosity, adults will judge as more numerous the dot pattern that occupies the larger space (Allik and Tuulmets, 1991).

In children, the first observation by Piaget (1952; Piaget and Inhelder, 1969) of the relationship between density and number was found in conservation tasks. Children 6–7 years of age were shown two lines of objects – eggs and egg cups – placed in one-to-one correspondence with equal inter-stimulus distances. After stipulation that the sets contained the same number, the children saw one set spatially transformed: the distance between the egg cups was increased. The children then saw the lengthened set as containing more. Piaget drew a few conclusions: children understand spatial displacement as a dimension relevant to number, and children do not understand that number is invariant, that physical attributes of sets are irrelevant to the (abstract) computation of number (Piaget and Inhelder, 1969; Gelman and Baillargeon, 1983). These observations by Piaget, together with the evidence from the adult literature on the perception of number and density, lead to the conclusion that, the more spread items are, the more likely we are to perceive number as “more.”

Speculations on the basis of experiments with animals, however, are driven by another set of findings. Quantity assessment of food items in patches of potential foraging by animals have been observed and analyzed by theories of optimal foraging (MacArthur and Pianka, 1966; Pyke et al., 1977; Stephens and Krebs, 1986) which predict that animals go for more. Animals evolved foraging strategies that maximize their net energy gain when foraging, namely, the energetic profit when foraging exceeds the energetic loss during foraging.

Suppose you are a baboon. You’re hungry. In the Namibian desert, food is scarce. You need to be selective where you forage in order not to waste energy and die. You strike lucky, and find two bushes of edible fruit to be harvested from. It is a simple matter: you’ll go for the bush containing more items. Evidence for non-human animal (monkeys: Hauser et al., 2000; horses: Uller and Lewis, 2009; salamanders: Uller et al., 2003) and human baby (Feigenson et al., 2002) selection of the larger of two sets is widespread. Now suppose, theoretically, that the shrubs you are assessing as possible feeding sources contain the same number of food items of same size. Which bush will you choose: the one in which the fruit are spread apart, or the one in which the fruit are close together? One of the chief constructs of Optimal Foraging models concerns energy expenditure. Marginal Value Theorem (Charnov, 1976) predicts when an animal should move to a new patch based on rate of return. There is a point when an animal would spend more energy searching for the next item within its current patch than it would to physically move to a new patch containing more items. Rate of return is correlated with the density of items within a patch. The closer packed items are within a patch, the less energy the individual will spend moving between the items. It is theoretically possible that the net gain of energy for a patch containing more items could be less than for a patch containing fewer items, if those items were spaced so widely apart that the animal wasted energy traveling between them. It would be highly adaptive for an individual to be sensitive not only to the number of items within a patch but also their relative density.

These two theoretical constructs – theories of numerosity perception in adults and Piagetian assessments in conservation tasks, versus optimal foraging theory – generate conflicting predictions. Optimal Foraging Theory would predict a choice for *more* – the closer together items are (more dense), the less energy an individual has to expend in gathering them up. Piagetian Theory and Theories of Adult Perceived Numerosity would also predict a choice for *more* – but here, the individual would be equating more distant with more (less dense).

It is clear, however, that there may be a difference between the numerical representation of objects (dots on a page/slide) and the numerical representation of edible items, as is the case of optimal foraging. And indeed, research with infants seems to indicate that the domain of food might be uniquely understood (Shutts et al., 2009). Recently, Van Marle and Wynn (2011) showed that infants will compare quantities of a food substance differing by a 1:4 ratio only when they can use density as a cue. This is preliminary evidence that infants use inter-stimulus distance to assess quantity.

In addition, animals seem to prefer higher density in sets of objects. Stevens et al. (2007) tested monkeys in conditions where only the density of the food items varied, not number. The results showed that they had a preference for a more dense set. These preliminary results suggest that both babies and monkeys may be sensitive to the distance between food items in a set. We set out to investigate this hypothesis with infants in the present study.

## Experiment 1

### Methods

#### Participants

Twenty (12 ♀) full-term infants participated in the study (Mean age = 10 months, 10 days; range 10;01–10;29). Five additional infants were excluded from the sample because of fussiness, namely, the infants did not make a choice during the familiarization phase. Participants were recruited as volunteers in the Essex/Suffolk/Cambridgeshire area through advertisements and were taken to the baby lab by their parent or caregiver.

#### Materials

The stimuli used for testing were *Sainsbury’s Economy*™ chocolate chip cookies measuring 50 mm in diameter. The trays used to display the cookies measured 300 mm × 290 mm and were made of dark gray sheet of metal. The four cookies were fixed to the trays using a combination of glue and *Blu tack* adhesive. They were laid out on each tray in a square configuration, so that each cookie formed one of the four edges of the square. The density of each set was determined by the distance between the cookies that formed the square. The inter-stimulus distance between the inner edges of the cookies was 40 mm (1 = more dense) and 160 mm (4 = less dense, ratio = 1:4). The “squares” were positioned so that they radiated out from the bottom inner corner of each tray. This was done to make the distance from the infant to the closest cookie on each tray the same for both sets. Neutral colored tea-towels covered the trays during the familiarization phase, so that the infant would not see the stimuli before the test phase. The display table measured 800 mm × 1200 mm. It was covered with a plain beige plastic tablecloth and was located in the center of the room. The testing room measured 1800 mm × 1800 mm, had white walls and was lit by an overhead halogen tube. It was empty apart from materials of the testing.

#### Design

The more dense array was presented on the left for half the babies, and on the right for the other half. Choice was coded as the set that the infant pointed to or touched. A testing session was considered over when (a) the infant made a choice by either touching one of the four stimuli or the tray on a clear reach to other (rather than the other) side, (b) the infant did not reach for or point to either tray after 60 s, or (c) if the infants choice was unclear, for example, if the infant simultaneously reached for or pointed to both trays. Infants were excluded when (b) and (c) were the case.

#### Procedure

The infants sat in the caregiver’s lap facing the experimenter and the stimuli. They were positioned so that their hands rested on the edge of the table. Throughout the experiment, babies were shown toys and edible items. They were allowed to chew on chewable toys shown to them, but they were not allowed to eat the cookies.

##### Familiarization phase

The experiment began with a pre-testing familiarization phase. The purpose of this was to (1) get the infant used to reaching for items on a tray and (2) familiarize the infant with the experimental stimuli. The familiarization phase began with the experimenter placing a toy onto an empty tray (identical to the trays the stimuli were presented on) and saying “Look [baby’s name]! Look at this! Would you like to pick it up?” This process was repeated until the infant readily picked up toys (ball, plastic keys, cup) from the tray. Following this, the experimenter hid the toys behind her back and picked and placed one of the cookies onto the tray saying “What’s this? You haven’t seen one of these before! Would you like to have a look?” As soon as the infant picked up the cookie and examined it, the caregiver was instructed to take the cookie from the infant and pass it back to the experimenter. Both cookie and tray were hidden underneath the table immediately thereafter.

If the infant did not respond after 30 s, having made no attempt to reach for the toys, the experimenter provided verbal encouragement: “Here, baby, would you like to grab it for me?” and would simultaneously draw the infant’s attention to the tray whilst speaking. If the infant did not respond after a further 30 s the trial was terminated.

##### Test phase

The test phase began directly after the removal of the cookie. The experimenter would say to the infant “Here, baby, we have some more cookies to play with!” The experimenter then uncovered both trays simultaneously. Following this, the experimenter simultaneously displayed both trays in a vertical position before pushing them within reaching distance of the infant. The experimenter and the caregiver would immediately avert their gaze downwards with a neutral facial expression until the infant reached for one of the trays. This method is generally used in infancy research (e.g., Feigenson et al., 2002), and has proven to be adequate for this kind of experiment. In a control experiment (not reported here) we contrasted this method with one in which parents/caregivers were blind-folded. No differences in result were observed.

The trial was terminated as soon as the infant hand made contact with one of the cookies on either tray. All trials were recorded with a *Sony DCR-TRV 900E* digital video camera for blind independent coding. The online data were recorded on a pre-printed record sheet by the experimenter following the trial.

### Results

Data from 17 infants who reached for either the more dense (1) or less dense (4) set were coded as choice. Thirteen infants selected the more dense set and four infants selected the less dense set. A binomial test revealed a significant difference between the two choices, *p* = 0.049, two-tailed.

### Discussion

The results of this experiment indicate that 10-month-old infants preferred to select the more dense set of four items. There are two interpretations for this finding. One is that human babies have an intrinsic natural propensity to go for more (density) in sets, namely, babies, like other non-human animals, prefer sets that are more compact, in which items are closer together.

Another interpretation is that 10-month-old infants prefer the more dense set because they equated the greater inter-stimulus distance in the less dense display with the impossibility of getting all the cookies at once with one hand (“all in one go” hypothesis), which could easily be done in the more dense set. Although we see this alternative too as part of the intrinsic preference to “go for more dense,” it could also be considered a strategy. To test the latter hypothesis, we decided to run another group of 10-month-old infants in Experiment 2.

## Experiment 2

The aim of Experiment 2 was to investigate whether the result of Experiment 1 was due to (1) an intrinsic preference to “go for more dense,” or (2) a strategy to select the set with items closer together (select all in one go hypothesis).

In order to address this possibility, we reduced the size of the stimuli while keeping inter-stimulus distance the same. By reducing the size of the stimuli we enable both sets to be kept within grabbing distance for a 10-month-old. That is, a 10-month-old hand would be able to grab all four stimuli at once whether the intra-stimulus distance was small or large. The inter-stimulus distance ratio (1:4) was kept the same.

### Method

The method was the same as in Experiment 1, except as follows.

### Participants

Eighteen (8 ♀) full-term infants participated in the study (Mean age = 10 months, 16 days; range 10;03–10;28). Four additional infants were excluded from the sample due to fussiness/no reach. Participants were recruited in the Essex/Suffolk/Cambridgeshire area through advertisements and were taken to the Lab by their parent or caregiver.

#### Materials

The food stimuli used were *Galaxy Minstrels*™. They measured 15 mm in diameter. The four candies were laid out in the same configuration as in Experiment 1 with a distance ratio of 1:4. The inter-stimulus distance between the inner edges of the candies was 10 mm (more dense) and 40 mm (less dense).

#### Design

Side of density ratio (1-L, 1-R) was counterbalanced across participants. The infant’s choice recorded as one level of density or the other (1 or 4). A testing session was considered over when the infants had made a choice. If the infant did not reach for or point to either tray, or if the infants choice was unclear (e.g., if the infant simultaneously reached for or pointed to both trays), this was coded as “no choice” and the participant was excluded.

### Results

Data from 16 infants who reached for either the more dense (1) or less dense (4) set were coded as choice. Thirteen infants selected the more dense set and three infant selected the less dense set. A binomial test revealed a significant difference, *p* = 0.020, two-tailed.

### Discussion

The findings in Experiment 2 supports the proposal that infants selected the more dense set because there is some mechanism at play that makes them prefer higher density than lower density. There is no evidence for the strategy explanation, whereby infants in Experiment 1 selected the more dense set because it was the set that enabled them to grab all four items at once as opposed to the less dense set, in which the four cookies were too far apart to grab all at once. Together, the results from Experiments 1 and 2 suggest that density assessment of sets of equal numerosity may be determined by a preference for things that are closer together.

## General Discussion

The present experiments were developed to start to address questions involving a variable that is generally conflated with numerical assessment and controlled for – set density. We contrasted two hypotheses in the fields of human perception and development (theories of numerosity perception in adults and Piaget’s number conservation ideas) and animal behavioral ecology (optimal foraging ideas). Evidence with children (Piaget’s conservation tasks) and adults (literature on numerosity perception) suggests that we tend to equate more as “more length” or “more space” in between items, while Optimal Foraging Theory predicts that patches of food containing items more packed together yield a better rate of return: animals seem to engage in evaluative computations which enable them to maximize profit and minimize cost. Although tentative and speculative at this stage, we pitted these two frameworks against each other because they predict opposite behaviors – children may prefer more as in *bigger* inter-stimulus distance, while animals may prefer more as in *smaller* inter-stimulus distance.

We set out to test these alternatives with two experiments addressing the question of inter-stimulus assessment in visual displays. Our results in Experiment 1 showed that, at 10 months, infants selected the more dense set by touching the chosen set. That is, infants reached and made a choice for the set in which elements were displayed closer together at the ratio of 1:4. Infants at 10 months make use of the variable “density” to make a numerical choice. This evidence supports preliminary evidence to show that infants have the ability to assess and compare quantities of a food substance (Van Marle and Wynn, 2011).

The alternative interpretation for the results of Experiment 1, that 10-month-old infants preferred the more dense set because they equated the smaller inter-stimulus distance in the more dense display with the possibility of getting all the cookies at once with the hand was addressed in Experiment 2. Two sets holding a 1:4 ratio between them, both with close enough items to be grabbed with one hand at once, were used in Experiment 2. Ten-month-old infants showed a preference for the more dense set, even though both sets could be grabbed with one hand at once. The preference observed in Experiment 1, therefore, cannot be attributed to a preference based on a “grab all in one go” strategy. Altogether, our results provide novel evidence that infants make decisions on numerical choices taking into account inter-stimulus distance (set density). The fact that the infants were not random in their choices means that this variable plays a role in numerical assessment.

A second conclusion stemming from these experiments is that there is a predisposition in young infants to select sets that contain items closer together. The data suggest that, at 10 months, infants are equipped with a capacity to discriminate two sets of equal number. What is even more extraordinary is that not only do infants detect the differences in density, but also make a particular choice, and reach for it. It is possible that these choices may apply uniquely to “foraging” situations, and indeed, the domain of food (Shutts et al., 2009) may be a special one. Further studies will be required to shed light onto this possibility.

## Conflict of Interest Statement

The authors declare that the research was conducted in the absence of any commercial or financial relationships that could be construed as a potential conflict of interest.
